# Early Gestational Wildfire-Related PM_2.5_ Exposure Is Associated with Lung Function in Offspring of Mothers with Asthma

**DOI:** 10.3390/ijerph23030314

**Published:** 2026-03-03

**Authors:** Gabriela Martins Costa Gomes, Adam M. Collison, Vanessa E. Murphy, Bronwyn K. Brew, Paul D. Robinson, Geoffrey G. Morgan, Karthik Gopi, Peter G. Gibson, Wilfried Karmaus, Joerg Mattes

**Affiliations:** 1 School of Medicine and Public Health, The University of Newcastle, Newcastle, NSW 2308, Australia; gabriela.martins@newcastle.edu.au (G.M.C.G.); adam.collison@newcastle.edu.au (A.M.C.); vanessa.murphy@newcastle.edu.au (V.E.M.); bronwyn.brew@newcastle.edu.au (B.K.B.); 2 Asthma and Breathing Research Program, Hunter Medical Research Institute, The University of Newcastle, Newcastle, NSW 2308, Australia; peter.gibson@newcastle.edu.au; 3 Children’s Health and Environment Program, Child Health Research Centre, The University of Queensland, South Brisbane, QLD 4101, Australia; paul.robinson@uq.edu.au; 4 School of Public Health, Faculty of Medicine and Health, University of Sydney, Camperdown, NSW 2050, Australia; geoffrey.morgan@sydney.edu.au (G.G.M.); karthik.gopi@sydney.edu.au (K.G.); 5 Centre for Safe Air, NHMRC Centre of Research Excellence, University of Tasmania, Hobart, TAS 7005, Australia; 6 Healthy Lives and Environments, NSHRC Research Network, University of Canberra, Bruce, ACT 2617, Australia; 7 Respiratory & Sleep Medicine Department, John Hunter Hospital, Newcastle, NSW 2305, Australia; 8 School of Public Health, University of Memphis, Memphis, TN 38152, USA; 9 Paediatric Respiratory & Sleep Medicine Department, John Hunter Children’s Hospital, Newcastle, NSW 2305, Australia

**Keywords:** maternal asthma, PM_2.5_, prenatal exposure, respiratory function tests, wildfire smoke, asthma, lung development

## Abstract

**Highlights:**

**Public health relevance—How does this work relate to a public health issue?**

- Wildfire-related air pollution is an increasing global public health concern due to climate change, with pregnant women and infants representing particularly vulnerable populations.
- This study examines prenatal exposure to wildfire-related PM_2.5_ during a critical developmental window and its association with early-life lung function and subsequent respiratory health.

**Public health significance—Why is this work of significance to public health?**

- Early gestational wildfire-related PM_2.5_ exposure was associated with differences in tidal breathing patterns in infancy. Infant inspiratory flow measures were also associated with airway reactance and asthma outcomes at six years.
- These findings suggest early gestation may be an important exposure window and highlight the need to understand how environmental hazards intersect with maternal asthma in shaping child respiratory health.

**Public health implications—What are the key implications or messages for practitioners, policy makers and/or researchers in public health?**

- Pregnant women with asthma may represent a group at increased vulnerability to wildfire smoke exposure, warranting consideration in public health planning.
- These findings support the integration of environmental exposure assessment into maternal and child health research and inform policies aimed at reducing health impacts of climate-driven air pollution.

**Abstract:**

Background: Prenatal exposure to air pollutants may increase the risk of adverse respiratory outcomes, particularly in offspring of asthmatic mothers. Evidence on wildfire-related PM_2.5_ exposure during pregnancy remains limited. This study investigated associations between early gestational wildfire-related PM_2.5_ exposure, infant lung function, and respiratory outcomes at 6 years. Methods: Gestational wildfire-related PM_2.5_ exposure patterns were characterised using group-based trajectory modelling and linked to infant lung function outcomes. Infant respiratory measurements were obtained at six weeks of age during behaviourally defined quiet sleep using tidal-breathing flow–volume loops (TBFVL). Airway mechanics at six years were assessed by impulse oscillometry (IOS) following international guideline standards. Trajectory modelling of PM_2.5_ during gestation was conducted in SAS (PROC TRAJ); all additional statistical analyses were performed in Stata IC 16.1. Results: Increased mean tidal inspiratory flow (MTIF, beta coefficient [β]: 10.51 mL/s, 95% CI: 3.66 to 17.36, *p* = 0.003) and peak tidal inspiratory flow (PTIF, β: 12.49 mL/s, 95% CI: 2.48 to 22.51, *p* = 0.014) were observed in infants born to mothers with higher wildfire-related PM_2.5_ exposure during early gestation (*n* = 420; *n* = 411 not exposed, *n* = 9 exposed). β-coefficients from infant mixed models were then used as proxy indicators and applied in linear regression models and associated with higher reactance at 5 Hz frequency (*n* = 73) at 6 years of age (PTIF: β: 9.88 mL/s, 95% CI: 0.10 to 19.67, *p* = 0.048 and MTIF: β: 13.43 mL/s, 95% CI: 1.43 to 25.44, *p* = 0.029). PTIF was further associated with asthma diagnoses at 6 years (aOR: 1.36, 95% CI: 1.07 to 1.73, *p* = 0.012; *n* = 259; *n* = 116 asthma). Conclusion: Early gestational exposure to wildfire-related PM_2.5_ may be linked with altered respiratory patterns in infancy and differences in airway reactance during childhood. Findings also suggest a relationship with asthma risk, although mechanisms remain uncertain.

## 1. Introduction

Air pollution exposure adversely affects respiratory health across all age groups. It is a well-established risk factor for developing asthma, with both children and adults being susceptible [1,2]. Air pollution exposure is also a risk factor for asthma exacerbations in children, directly impacting lung function and respiratory symptoms [3,4,5]. Recent preschool studies further support these associations and highlight the contribution of combined particulate and gaseous pollutant exposures to asthma and rhinitis morbidity in early childhood [6]. Its effects can begin as early as prenatally [7], and evidence suggests that early impairment of lung function predicts later respiratory morbidity [8,9].

The connection between the mother and fetus plays a crucial role in respiratory disease development, aligning with the developmental origins of disease hypothesis, which suggests that early-life exposures can influence future disease susceptibility [10]. While the placenta acts as a barrier, it is not impenetrable and environmental air pollutant particles that cross the placental barrier can affect fetal development [11,12]. Fine particles can penetrate the alveolar region of the lungs through inhalation, enter the systemic circulation, and reach the placenta, potentially impacting the fetus [11].

Australia’s 2019–2020 wildfires were unprecedented in both scale and duration, burning over 17 million hectares, with New South Wales (NSW) experiencing the most extensive damage, affecting 5.5 million hectares of land over a period of months [13,14]. Air pollutants from wildfires are considered to significantly increase health risks, with fine particles with an aerodynamic diameter of less than 2.5 μm (PM_2.5_) being of particular concern as the most hazardous component of air pollution [15]. Wildfires lead to extremely poor air quality, with PM_2.5_ concentrations exceeding the 95th percentile of historical mean data [14]. Ambient PM_2.5_ exposure during gestation has been linked to altered fetal development and adverse postnatal health outcomes [16] and reduced lung function in children [17], supporting the notion that such exposure may negatively impact pulmonary development in utero. Recent large-scale cohort studies further suggest that early to mid-pregnancy represents a critical window of susceptibility to PM_2.5_ exposure, with exposure during this period associated with reduced birth weight, indicating heightened fetal vulnerability during early gestation [18].

In pregnant women, asthma itself increases the risk of adverse neonatal outcomes, including respiratory complications [19,20], and prenatal exposure to air pollutants has been associated with higher risks of conditions like transient tachypnoea of the newborn, asphyxia, and respiratory distress syndrome [21]. Although existing studies have explored the effects of prenatal air pollution exposure on infant and child lung function, no data are available on the impact of wildfire-related PM_2.5_ exposure during gestation in the offspring of asthmatic pregnant women [22]. During the 2019–2020 wildfires in Australia, we evaluated infant lung function and respiratory outcomes in a cohort of mothers with asthma. We therefore examined (i) whether early-gestation wildfire-related PM_2.5_ exposure was associated with infant tidal breathing measures, and, (ii) among participants with 6-year follow-up, whether infant tidal inspiratory flow measures were associated with later respiratory outcomes. We hypothesized that such exposure may be associated with altered early-life respiratory patters, and that infant-lung function differences may relate to later respiratory outcomes.

## 2. Materials and Methods

### 2.1. Study Participants

Pregnant women, 18 years or older, with physician-diagnosed asthma, were enrolled in the Breathing for Life Trial (BLT) (from Newcastle and Syndey, Australia) [23,24] at 12–22 weeks’ gestation (supported by ultrasound or clinical obstetric assessment). Gestational age at enrolment (and corresponding pregnancy onset) was determined from antenatal clinical records. Eligible participants completed an interviewer-administered questionnaire on sociodemographic and lifestyle factors, including age, ethnicity, parity, and health status. Enrolled mothers and their infants were invited for a follow-up visit when infants were 4 to 7 weeks old. Infant inclusion criteria for lung function testing required the absence of major birth defects or perinatal diseases that would prevent performing unsedated lung function tests. Infants who experienced respiratory illness in the two weeks prior to testing were rescheduled. A flow diagram is provided in the Supplementary Materials (Figure S1). All participants provided written informed consent before participation. The study was approved by the Hunter New England Local Health District Human Research Ethics Committee 2019/ETH03856.

### 2.2. Infant Lung Function

Lung function was conducted in unsedated infants during behaviorally defined quiet sleep. Testing was performed supine with an infant mask to maintain a tight seal (Homedica, Huenenberg, Switzerland), according to ERS/ATS standards [25,26]. Flow was measured using an ultrasonic flow meter (Spiroson; EcoMedics, Duernten, Switzerland). Data were collected with Spiroware 2 (EcoMedics AG, Dürnten, Switzerland).

Tidal breathing flow volume loops (TBFVLs) were measured for 90 s to obtain at least 30 good-quality breaths [26,27]. Analyses were performed using Wbreath (version 3.28.0; Ndd Medizintechnik, Zürich, Switzerland). Data were included only if there was no significant volume drift (<3 mL/s) after adjusting for environmental conditions, temperature, and mask dead space. Trials exceeding the drift threshold were assumed to contain a leak and excluded.

### 2.3. Child Asthma Outcomes

In an ongoing follow-up at 6 years of age, child asthma was reported by a parent or guardian using the International Study of Asthma and Allergies in Childhood (ISAAC) questionnaire [28] among children with available 6-year follow-up data (*n* = 259), all of whom were from the no-exposure group. The primary outcome, “ever asthma”, was defined as an affirmative response to the question, “Has your child been diagnosed with asthma by a doctor?”.

### 2.4. Impulse Oscillometry (IOS) Assessment

At 6 years, IOS was performed during quiet tidal breathing with the child seated upright, using the MasterScreen system (Jaeger Co., Ltd., Hochberg, Germany). Quality control followed ERS/ATS standards, requiring coherence ≥ 0.8 at 5 Hz and (CV) ≤ 10% across acceptable trials. Measurements with poor coherence or excessive variability were excluded [29]. Measurements were repeated until either three acceptable curves with CV ≤ 10%, or two with CV ≤ 5%, were obtained; those with the best coherence were used. IOS results were expressed as percent predicted, using Dencker reference data [30]. Resistance (Rrs) and reactance (Xrs) at 5 Hz were evaluated, with abnormal values defined as ≥1.65 standard deviations from predicted [31].

### 2.5. Air Pollution Assessment

Air pollution PM_2.5_ data, including seasonal trend information and statistical flags for identifying wildfire-related PM_2.5_, were obtained from the Centre for Safe Air (CSA), as described previously [32]. Data from the CSA’s National Air Pollution Monitor Database (NAPMD) [33], which integrates fixed and field monitors across Australian states and territories (2001–2020) with land use, weather, and satellite observations, were used to predict daily PM_2.5_ via a Random Forest Algorithm. Model performance and agreement with regulatory monitor PM_2.5_ measurements have been reported previously, including cross-validation against ambient monitoring data [34]. Daily exposure was estimated within a 5 km × 5 km grid around each woman’s residence. Residential addresses recorded in antenatal clinical records at enrolment were geocoded to latitude/longitude and assigned to the corresponding 5 km × 5 km grid cell; the daily predicted PM_2.5_ value for that grid cell was then linked to each participant for each day of pregnancy. Seasonal and trend decomposition with loess was applied to determine daily PM_2.5_ components during pregnancy [34].

Wildfire smoke-affected days were defined as those with total PM_2.5_: (1) above the 95th percentile or two standard deviations of historical daily concentrations, and (2) satellite confirmation of a wildfire within 50 km [32]. These criteria correspond to the “statistical flags” provided by CSA to identify smoke-affected days and isolate the wildfire-attributable PM_2.5_ component. Wildfire-specific daily PM_2.5_ was then calculated as the difference between absolute PM_2.5_ on wildfire days and the seasonal/trend components (representing the portion attributable to wildfire smoke, including controlled hazard reduction landscape burning). Participant residential address at enrolment and at infant visit were used for exposure assignment. Neonatal outcomes associated with prenatal wildfire smoke exposure in this cohort have been reported previously [35].

### 2.6. Statistical Analysis

To describe varying wildfire-related PM_2.5_ exposure during gestation, a semi-parametric group-based trajectory modelling (PROC TRAJ) approach was applied in SAS^®^ 9.4 (SAS Institute Inc., Cary, NC, USA) [36,37]. This combines latent growth curve and mixture modelling to identify distinct exposure trajectories [36]. Parameters were estimated by maximum likelihood with a binary logit model [38].

Models with 2–4 trajectories and linear, quadratic, or cubic terms were tested to best capture temporal variation. The model with the lowest Bayesian Information Criterion (BIC) was selected [39]. PROC TRAJ assumes data are missing completely at random. Individuals were assigned to the trajectory group with the highest posterior probability.

Descriptive statistics, mixed linear models, and regression analyses were performed using Stata IC 16.1 (Stata Corporation, College Station, TX, USA). Group differences were evaluated using Chi-square or *t*-tests or Mann–Whitney U test. Mixed linear models assessed our primary outcome: association of gestational exposure to wildfire-related PM_2.5_ with infant lung function. To account for non-independence of infants from the same pregnancy (twins), we included a random intercept for mother/pregnancy ID, using mixed models (using autoregressive covariance with restricted maximum likelihood). These were adjusted for sex, maternal smoking during pregnancy, maternal asthma exacerbation during pregnancy, weight at the time of infant lung function testing, breastfeeding at the time of infant lung function testing, and multiple births. Confounders were excluded if they influenced the estimate by <10% (socioeconomic status, inhaled corticosteroid (ICS) use during pregnancy, caesarean section, prematurity, age). Regression analyses then assessed our secondary outcome: the long-term impact of infant lung function parameters on respiratory outcomes at 6 years old (for which data collection is ongoing). Adjustments matched those above. Due to limited 6-year follow-up data for directly exposed children (COVID-19 restrictions), we could not directly assess associations with later outcomes. Instead, β-coefficients from our mixed model (quantifying gestational wildfire-related PM_2.5_ exposure related to infant lung function associations) were used as proxy exposure values in regression models at 6 years. Significance was set at *p* ≤ 0.05.

This study was reported in accordance with the Strengthening the Reporting of Observational Studies in Epidemiology (STROBE) guidelines.

## 3. Results

### 3.1. Study Population

From March 2013 to March 2020, 1264 pregnancies with available wildfire-related PM_2.5_ data were included in the exposure modelling (Figure S1). Group-based trajectory modelling identified three distinct exposure patterns across gestation (Figure S2). The majority of pregnancies (Group 1: *n* = 1211, 95.8%) had persistently low wildfire-related PM_2.5_ exposure (median (IQR) daily wildfire-related PM_2.5_: 7.3 (6.8–7.6) µg/m^3^). A second group (Group 2: *n* = 30, 3.4%) showed higher exposure during mid-to-late gestation (months 5–8; median (IQR): 17.4 (14.3–20.8) µg/m^3^), and a third group (Group 3: *n* = 23, 1.8%) had higher exposure during early gestation (months 3–4; (median (IQR): 24.7 (23.3–26.3) µg/m^3^).

For our primary outcome (infants’ lung function collected between May 2014 to November 2020), 603 infants attended follow-up, of whom 420 had valid tidal-breathing measurements and complete exposure data. These infants were assigned to maternal exposure trajectories. In this subset, only nine infants (2.1%) belonged to the early-exposure group (Group 3), and none were from the mid-to-late exposure group (Group 2). This absence resulted from limited availability of infants born during the period of wildfire activity and subsequent COVID-19- related disruptions that prevented follow-up. Therefore, Group 1 (*n* = 411, 97.9%) served as the reference for further analysis (Figure S1). Baseline characteristics can be found in Table 1.

### 3.2. Gestational Exposure to Wildfire-Related PM_2.5_ and Infant Lung Function (Primary Outcome)

Analyzing infant lung function baseline characteristics using a *t*-test, tidal volume (TV, *p* = 0.014), minute ventilation (V′E, *p* = 0.002), mean tidal expiratory flow (MTEF, *p* = 0.003), peak tidal expiratory flow (PTEF, *p* = 0.007), mean tidal inspiratory flow (MTIF, *p* = 0.008), and peak tidal inspiratory flow (PTIF, *p* = 0.032) were significantly higher among infants born to mothers with high wildfire-related PM_2.5_ exposure during early gestation, compared to those born to mothers with no exposure (Table 2).

To adjust for twin pairs, a mixed linear model was applied. Early gestational PM_2.5_ exposure remained associated with differences in infant tidal breathing. TV (β: 5.16 mL, 95% CI: 1.07 to 9.27, *p* = 0.013), V′E (β: 315.01 mL, 95% CI: 145.57 to 484.46, *p* = 0.0003), MTEF (β: 10.48 mL/s, 95% CI: 4.28 to 16.68, *p* = 0.001), PTEF (β: 16.10 mL/s, 95% CI: 6.65 to 25.55, *p* = 0.001), MTIF (β: 10.51mL/s, 95% CI: 3.66 to 17.36, *p* = 0.003), and PTIF (β: 12.49 mL/s, 95% CI: 2.48 to 22.51, *p* = 0.014) were increased in infants born to mothers with high wildfire-related PM_2.5_ exposure during early gestation (Table 3).

### 3.3. Infant Lung Function Parameters and Respiratory Outcomes at 6 Years of Age (Secondary Outcome)

Of the 420 infants with valid lung-function measurements, 73 children (17.4%) participated in the 6-year follow-up visit and completed impulse oscillometry (IOS), and 259 children (61.7%) had asthma outcomes available from the ISAAC questionnaire, of whom 116 (44.8%) were diagnosed with asthma (Table S1). No children in the early-exposure group contributed 6-year data, as these births coincided with periods affected by the 2019–2020 wildfires and the subsequent COVID-19 restrictions. As a result, direct analysis of prenatal wildfire-related PM_2.5_ exposure and outcomes at age six was not possible.

To explore whether infant tidal-breathing patterns were related to later airway mechanics, β-coefficients from the infant mixed models (representing the magnitude of association between early gestational PM_2.5_ exposure and infant lung-function measures) were used as proxy indicators. These proxy values were applied in linear regression models examining associations with IOS parameters at six years.

Higher infant inspiratory flow parameters remained associated with differences in airway reactance at school age. Both MTIF (β: 13.43 mL/s, 95% CI: 1.43 to 25.44, *p* = 0.029) and PTIF (β: 9.88 mL/s, 95% CI: 0.10 to 19.67, *p* = 0.048) were positively associated with higher reactance 5 Hz (Table S2).

Associations with asthma outcomes were assessed using logistic regression among children with both infant lung-function and ISAAC data (*n* = 259). Higher infant PTIF remained associated with greater odds of asthma diagnosis at age six (aOR 1.36, 95% CI: 1.07–1.73; *p* = 0.012), with each 1 mL/s increase in PTIF at six weeks corresponding to an increase in the odds of asthma at six years (Table S2).

## 4. Discussion

To the best of our knowledge, this is the first study to examine early gestational exposure to wildfire-related PM_2.5_ in pregnancies complicated by maternal asthma and its association with infant tidal-breathing patterns and later childhood respiratory outcomes. Infants exposed during early gestation (daily: 23.3–26.3 µg/m^3^) demonstrated higher tidal volumes and higher inspiratory and expiratory flow parameters at six weeks of age. These changes, particularly increased PTIF and MTIF, were associated with higher reactance at 5 Hz at 6 years is age and PTIF was also associated with higher odds of asthma diagnosis. These observed differences may reflect alterations in early respiratory behaviour, although they should be interpreted as associations rather than evidence of a specific underlying physiological mechanism.

Early gestation corresponds to a period of rapid lung development, and environmental exposures during this window may influence later respiratory patterns. Environmental chemicals can disrupt key signaling pathways in lung morphogenesis, affecting both branching and alveolar development [40]. Experimental studies suggest that PM_2.5_ exposure reduces alveolar number [41] and impairs alveolarization [42]. Support for the importance of this early gestational window also comes from large population-based studies demonstrating that PM_2.5_ exposure during early to mid-pregnancy is associated with reduced birth weight, a marker of disrupted fetal development and placental function [18]. Maternal asthma has also been independently linked to placental complications and low birth weight, and evidence shows that impaired intrauterine growth is associated with altered lung development and persistent reductions in lung function later in life [43]. Together, these findings support the concept that disruptions to fetal growth during critical developmental windows may have lasting consequences for lung structure and function.

The higher tidal volumes and flow rates observed in this study may reflect one manifestation of such developmental effects, though other influences such as chest wall compliance or breathing variability cannot be excluded. Since respiratory rate was unchanged, this pattern reflects deeper breaths (higher tidal volume) with higher inspiratory flow, increasing minute ventilation; this could reflect altered mechanics (compliance/elastic recoil or lung volume recruitment) and/or altered neural control of breathing (ventilatory drive and respiratory muscle recruitment) [44]. Further, no previous research has evaluated wildfire exposure during pregnancy in women with asthma and its later influence on infant lung health. Wildfire smoke may increase maternal inflammation and impair placental function [22,45,46] both of which heighten fetal vulnerability in pregnancies complicated by asthma.

These results align with prior evidence linking early lung impairment to later respiratory risk [47,48,49]. Deficits in infant lung function, including tidal breathing indices, have been linked to later asthma risk [50]. Further, prenatal air pollution exposure has been associated with reduced childhood lung function [51,52] and higher risk of wheezing and asthma [42]. Abnormal tidal breathing parameters, such as elevated PTIF, have also been reported in infants with acute viral bronchiolitis admitted to the ICU [53]. In our study, higher PTIF in infancy was associated with prenatal wildfire-related PM_2.5_ exposure and also associated with asthma at age six. Because no exposed infant contributed to the six-year follow-up, these associations reflect relationships between early infant physiology and later outcomes rather than direct effects of prenatal wildfire exposure. Although tPTEF/tE, a timing index sensitive for expiratory flow limitation, was not significantly different, PTIF, a magnitude-based measure of inspiratory flow, showed consistent associations. This pattern may be consistent with effects on compliance and elastic recoil rather than airway obstruction.

Infant PTIF was also associated with asthma diagnosis at age six. This suggests a possible link between early tidal-breathing characteristics and later respiratory morbidity. However, asthma is multifactorial, and causal inference cannot be made from these results. Our findings highlight the need for longitudinal lung function measures to clarify pathways from prenatal exposure to long-term respiratory outcomes. We hypothesize that in utero exposure to wildfire-related PM_2.5_ may influence branching morphogenesis and alveolarization, potentially contributing to early-life breathing adaptations and later differences in airway mechanics.

The mechanisms by which exposure to wildfire smoke affects infant lung function and later outcomes are not well established. Recent multi-omics studies suggest that the maternal metabolic environment during pregnancy (including microbiome-derived and circulating metabolite) may influence fetal immune development and shape offspring susceptibility to atopic disease [54]. Air pollution exposure during pregnancy has been shown to impact the cord blood environment. Studies demonstrate that exposure to polycyclic aromatic hydrocarbons or PM_2.5_ during early pregnancy modify lymphocyte immunophenotypes, potentially disrupting the Th1/Th2 balance and contributing to immune dysregulation [55]. Other studies report shifts in cord blood cell populations linked to inflammatory respiratory diseases in childhood [56].

It is important to note that follow-up in this cohort was limited by contextual factors. Australia’s 2019–2020 wildfires coincided with holiday periods and the COVID-19 lockdown, restricting data collection, particularly for later-pregnancy exposures (months 5–9). Only a subset of the cohort underwent lung function testing, with 420 infants completing tidal breathing analysis and 73 children completing IOS assessments; this reduced the effective sample size in the exposure-defined groups. Despite reduced statistical power, associations were detectable in adjusted models; however, findings should be interpreted cautiously. Postnatal PM_2.5_ exposure was not available for adjustment; however, exposures were defined during pregnancy independent of the lung function, and any residual postnatal confounding would most likely attenuate associations based on ambient prenatal estimates. Maternal asthma severity was assessed using recorded asthma exacerbations during pregnancy as the closest available indicator of severity in this cohort. In addition, the independent definition of PM_2.5_ exposure resulted in only a small sample of exposed children (*n* = 9); however, these children were part of a larger exposed group with other adverse health markers [35]. Hence, larger studies are needed to better understand the impact of wildfire exposure across pregnancy stages, especially among mothers with asthma. Importantly, none of the children included in the 6-year follow-up belonged to the wildfire-exposed groups; therefore, surrogate exposure estimates were applied to explore whether infant lung function differences predicted later outcomes. While a limitation, this approach also provided a strength by allowing us to extend the analysis beyond directly exposed participants and evaluate whether early-life lung function alterations, quantified by β-coefficients, were predictive of later respiratory outcomes. This strengthened the biological plausibility of our findings by linking prenatal exposure, infant lung function, and school-age outcomes within a single framework, despite follow-up restrictions imposed by the pandemic. Of note, the use of β-coefficients as proxy indicators should be interpreted as exploratory rather than confirmatory.

## 5. Conclusions

This study suggests that early gestational exposure to wildfire-related PM_2.5_ in pregnancies complicated by maternal asthma is associated with measurable alterations in infant lung function, specifically higher inspiratory flows at 6 weeks of age. Infant inspiratory flow measures were associated with airway reactance at 6 years and with asthma diagnosis in childhood, indicating that prenatal exposure may disrupt critical stages of lung development and trigger compensatory breathing adaptations that contribute to long-term adverse respiratory outcomes. These findings should be interpreted as associations rather than evidence of a specific physiologic mechanism. Understanding these effects is critical for developing targeted interventions and public-health guidelines, particularly for pregnant women with pre-existing respiratory conditions who may be especially vulnerable to wildfire smoke.

## Figures and Tables

**Table 1:** Baseline characteristics of study infants, stratified by maternal wildfire-related PM_2.5_ exposure during the first four months of gestation.

	Total *n* = 420		
	High Exposure During Early Gestation (*n* = 9)	No Exposure During Gestation (*n* = 411)	*p* Value
Asthma exacerbation during pregnancy *n* (%)	3 (33.3)	92 (22.4)	0.437
Maternal smoking during pregnancy *n* (%)	0 (0.0)	57 (13.9)	0.229
Preterm birth *n* (%)	0 (0.0)	34 (8.3)	0.368
Caesarean section *n* (%)	2 (22.2)	131 (31.9)	0.538
Male *n* (%)	6 (66.7)	214 (52.1)	0.386
Twins *n* (%)	2 (22.2)	6 (1.7)	0.00001
SEIFA (IRSD) quintiles, *n* (%)			0.327
1 (most disadvantaged)	2 (22.2)	46 (11.2)	
2	4 (44.4)	94 (22.9)	
3	2 (22.2)	163 (39.7)	
4	1 (11.1)	51 (12.4)	
5 (least disadvantaged)	0 (0.0)	57 (13.9)	
Gestational age at birth in weeks *	38.7 (2.0)	39.1 (1.6)	0.447
Birth weight in kg *	3.2 (0.7)	3.4 (0.6)	0.317
Age at infant lung function test in weeks *	6.7 (1.3)	6.6 (1.9)	0.919
Weight at infant lung function test in kg *	4.9 (0.8)	4.9 (0.7)	0.930
Length at infant lung function test in cm *	57.2 (1.7)	56.0 (3.2)	0.306
Breastfeeding at infant lung function test date *n* (%)	7 (77.8)	293 (71.3)	0.670

Groups were compared using either a *t*-test or a chi-square test as appropriate. * values show mean (SD)—*p*-values from unadjusted comparisons and do not account for clustering within multiple (twin) pregnancies. SEIFA, socio-economic indexes for areas. IRSD, Index of Relative Socioeconomic Disadvantage.

**Table 2:** Baseline characteristics of infant lung function parameters, stratified by maternal wildfire-related PM_2.5_ exposure during the first four months of gestation.

	Total *n* = 420		
	High Exposure During Early Gestation (*n* = 9)	No Exposure During Gestation (*n* = 411)	*p* Value
TV, mL/kg	8.0 (1.4)	6.9 (1.3)	**0.019**
V′E, mL/kg	365.7 (71.5)	303.2 (57.8)	**0.006**
RR, min	46.0 (5.5)	45.0 (10.2)	0.516
MTEF, mL/s/kg	11.0 (2.4)	8.9 (2.1)	**0.007**
PTEF, mL/s/kg	16.3 (3.9)	13.3 (3.2)	**0.013**
MTIF, mL/s/kg	13.9 (2.7)	11.9 (2.3)	**0.012**
PTIF, mL/s/kg	19.0 (3.6)	16.6 (3.3)	**0.027**
tPTEF/tE, %	30.0 (4.7)	31.7 (9.7)	0.780

Values show mean (SD). Mann–Whitney U test used to compare groups. *p* value < 0.05 in bold. TV, tidal volume; V′E, minute ventilation; RR, respiratory rate; MTEF, mean tidal expiratory flow; PTEF, peak tidal expiratory flow; MTIF, mean tidal inspiratory flow; PTIF, peak tidal inspiratory flow; tPTEF/tE, time to peak tidal expiratory flow divided by total expiratory time.

**Table 3:** Mixed linear model to assess the association of wildfire-related PM_2.5_ with infant lung function.

	Total Cohort (*n* = 420) *n* = 9 Infants in High Exposure Group During Early Gestation *n* = 411 Non-Exposed			
Gestational Wildfire-Related PM_2.5_	Crude Analysis		Multivariable Analysis *	
	Coefficient (95% CI)	*p* Value	Coefficient (95% CI)	*p* Value
TV, mL	6.23 (1.52 to 10.9)	**0.010**	5.16 (1.07 to 9.27)	**0.013**
V′E, mL	337.12 (152.49 to 521.74)	**0.0003**	315.01 (145.57 to 484.46)	**0.0003**
RR, min	1.00 (−5.79 to 7.79)	0.773	1.52 (−5.23 to 8.26)	0.659
MTEF, mL/s	10.98 (4.49 to 17.46)	**0.001**	10.48 (4.28 to 16.68)	**0.001**
PTEF, mL/s	16.58 (6.81 to 26.34)	**0.001**	16.10 (6.65 to 25.55)	**0.001**
MTIF, mL/s	11.68 (4.12 to 19.23)	**0.002**	10.51 (3.66 to 17.36)	**0.003**
PTIF, mL/s	14.07 (3.16 to 24.97)	**0.011**	12.49 (2.48 to 22.51)	**0.014**
tPTEF/tE, %	−2.06 (−8.66 to 4.54)	0.540	−2.01 (−8.55 to 4.52)	0.546

* Analysis adjusted for sex, maternal smoking during pregnancy, maternal asthma exacerbation during pregnancy, weight at time of infant lung function testing, breastfeeding at time of infant lung function testing, multiple births (autoregressive covariance with restricted maximum likelihood). *p* value < 0.05 in bold. TV, tidal volume; V′E, minute ventilation; RR, respiratory rate; MTEF, mean tidal expiratory flow; PTEF, peak tidal expiratory flow; MTIF, mean tidal inspiratory flow; PTIF, peak tidal inspiratory flow; tPTEF/tE, time to peak tidal expiratory flow divided by total expiratory time.

## Data Availability

All data relevant to the study are included in the article or provided as Supplementary Information. Data supporting the findings of this study are available from the corresponding author upon reasonable request.

## Supplementary Materials

The following supporting information can be downloaded at: https://www.mdpi.com/article/10.3390/ijerph23030314/s1. Figure S1: Flow diagram of inclusion and exclusion criteria for the study cohort; Figure S2: Patterns of cumulative PM_2.5_ exposure from wildfire days by month across the 9 months of gestation; Table S1: Baseline characteristics of study children at 6 years of age included in an additional analysis of infant lung function and later respiratory outcomes; Table S2: Regression analysis to assess the effects of infant lung function on later respiratory outcomes at 6 years old.

## Abbreviations

The following abbreviations are used in this manuscript:

BLT	Breathing for Life Trial
COPD	Chronic Obstructive Pulmonary Disease
ERS/ATS	European Respiratory Society/American Thoracic Society
Hz	Hertz
ICU	Intensive Care Unit
IOS	Impulse Oscillometry
ISAAC	International Study of Asthma and Allergies in Childhood
MTIF	Mean Tidal Inspiratory Flow
MTEF	Mean Tidal Expiratory Flow
PM_2.5_	Particulate Matter < 2.5 micrometers
PTIF	Peak Tidal Inspiratory Flow
PTEF	Peak Tidal Expiratory Flow
RR	Respiratory Rate
Rrs	Respiratory System Resistance
TBFVL	Tidal Breathing Flow–Volume Loop
Th1/Th2	T-helper Cell Type 1/Type 2
tPTEF/tE %	time to Peak Tidal Expiratory Flow divided by Total Expiratory time
TV	Tidal Volume
V′E	Minute Ventilation
Xrs	Respiratory System Reactance
