# Supplementary material for: Early Gestational Wildfire-Related PM2.5 Exposure Is Associated with Lung Function in Offspring of Mothers with Asthma
Source: Int J Environ Res Public Health. 2026 Mar 3;23(3):314. doi: 10.3390/ijerph23030314 (PMC13026569; doi:10.3390/ijerph23030314)
Supplement: Supplementary file 1 [file ijerph-23-00314-s001.zip › supp data/Table S2.pdf]

	Rrs at 5Hz<br>(n = 73)<br>Coefficient (95% CI)<br>p value		Xrs at 5Hz<br>(n = 73)<br>Coefficient (95% CI)<br>p value		Asthma outcome<br>(n = 259 total, 116 with asthma)<br>OR (95% CI)<br>p value	
	**Crude analysis**	**Multivariable analysis***	**Crude analysis**	**Multivariable analysis***	**Crude analysis**	**Multivariable analysis***
TV, mL	-2.20 (-8.50 to 4.10)<br>0.489	-0.74 (-6.80 to 5.32)<br>0.808	3.44 (-6.39 to 13.27)<br>0.488	0.68 (-8.77 to 10.14)<br>0.885	1.25 (1.00 to 1.56)<br>**0.048**	2.14 (1.00 to 1.54)<br>0.051
V'E, mL	-2.58 (-10.16 to 5.00)<br>0.499	-2.20 (-10.07 to 5.68)<br>0.580	10.61 (-1.00 to 22.21)<br>0.073	7.67 (-4.51 to 19.84)<br>0.213	1.23 (0.90 to 1.69)<br>0.200	1.15 (0.83 to 1.60)<br>0.402
MTEF, mL/s	-3.53 (-10.29 to 3.23)<br>0.301	-3.11 (-9.97 to 3.76)<br>0.369	4.92 (-5.64 to 15.49)<br>0.356	2.76 (-7.99 to 13.51)<br>0.610	1.05 (0.78 to 1.40)<br>0.759	1.01 (0.75 to 1.36)<br>0.949
PTEF, mL/s	-1.31 (7.91 to 5.30)<br>0.694	-0.99 (-7.72 to 5.73)<br>0.770	6.65 (-3.55 to 16.85)<br>0.198	5.22 (-5.20 to 15.64)<br>0.321	1.19 (0.89 to 1.60)<br>0.234	1.16 (0.86 to 1.57)<br>0.335
MTIF, mL/s	0.44 (-7.01 to 7.88)<br>0.907	1.18 (-6.80 to 9.16)<br>0.769	15.59 (4.57 to 26.61)<br>**0.006**	13.43 (1.43 to 25.44)<br>**0.029**	1.37 (1.03 to 1.80)<br>**0.028**	1.28 (0.96 to 1.70)<br>0.089
PTIF, mL/s	1.92 (-4.15 to 8.00)<br>0.530	2.60 (-3.83 to 9.03)<br>0.422	11.96 (2.88 to 21.03)<br>**0.011**	9.88 (0.10 to 19.67)<br>**0.048**	1.39 (1.10 to 1.76)<br>**0.006**	1.36 (1.07 to 1.73)<br>**0.012**

*Analysis adjusted for sex, maternal smoking during pregnancy, maternal asthma exacerbation during pregnancy, weight at time of infant lung function testing, breastfed until infant lung function testing. Results estimated based on the effect size observed in exposed children. p value < 0.05 in bold.
OR, odds ratio; TV, tidal volume; V'E, minute ventilation; MTEF, mean tidal expiratory flow; PTEF, peak tidal expiratory flow; MTIF, mean tidal inspiratory flow; PTIF, peak tidal inspiratory flow; Rrs, resistance; Xrs, reactance; Hz, Hertz; aOR, adjusted odds ratio.
