# Supplementary material for: Early Gestational Wildfire-Related PM2.5 Exposure Is Associated with Lung Function in Offspring of Mothers with Asthma
Source: Int J Environ Res Public Health. 2026 Mar 3;23(3):314. doi: 10.3390/ijerph23030314 (PMC13026569; doi:10.3390/ijerph23030314)
Supplement: Supplementary file 1 [file ijerph-23-00314-s001.zip › supp data/Table S1.pdf]

	6 years IOS assessment and infant lung function data n = 73	6 years ISAAC questionnaire and infant lung function data n = 259
Asthma exacerbation during pregnancy *n (%)*	25 (34.3)	66 (25.5)
Maternal smoking during pregnancy *n (%)*	11 (15.1)	29 (11.2)
Preterm birth *n (%)*	9 (12.3)	22 (8.5)
Caesarean section *n (%)*	24 (32.9)	86 (33.2)
Male *n (%)*	41 (56.2)	138 (53.3)
Twins *n (%)*	2 (2.7)	2 (0.8)
Gestational age at birth in weeks*	38.9 (1.7)	39.1 (1.6)
Birth weight in kg*	3.4 (0.6)	3.4 (0.6)
**Infant assessments**		
Age at infant lung function test in weeks*	6.0 (1.7)	6.6 (1.9)
Weight at infant lung function test in kg*	4.8 (0.7)	4.9 (0.7)
Length at infant lung function test in cm*	55.6 (2.6)	56.0 (3.1)
Breastfed until infant lung function test date *n (%)*	53 (72.6)	192 (74.1)
**6 years assessments**		
Asthma diagnoses until test date *n (%)*	37 (50.6)	116 (44.8)
Age at lung function test in years*	7.2 (0.6)	-
Weight at lung function test in kg*	27.6 (6.4)	-
Length at lung function test in cm*	125.8 (6.5)	-

ISAAC, International Study of Asthma and Allergies in Childhood
*values show mean (SD)
