# Supplementary material for: Early Gestational Wildfire-Related PM2.5 Exposure Is Associated with Lung Function in Offspring of Mothers with Asthma
Source: Int J Environ Res Public Health. 2026 Mar 3;23(3):314. doi: 10.3390/ijerph23030314 (PMC13026569; doi:10.3390/ijerph23030314)
Supplement: Supplementary file 1 [file ijerph-23-00314-s001.zip › supp data/4.SuppData_PM2.5 and lung function_ IJERPH.pdf]

Article

Early gestational wildfire-related $PM_{2.5}$-exposure is associated with lung function in offspring of mothers with asthma

Gabriela Martins Costa Gomes, Adam M Collison, Vanessa E Murphy, Bronwyn K Brew, Paul D Robinson, Geoffrey G Morgan, Karthik Gopi, Peter G Gibson, Wilfried Karmaus, Joerg Mattes

Citation: To be added by editorial staff during production.

Online Supplement Data

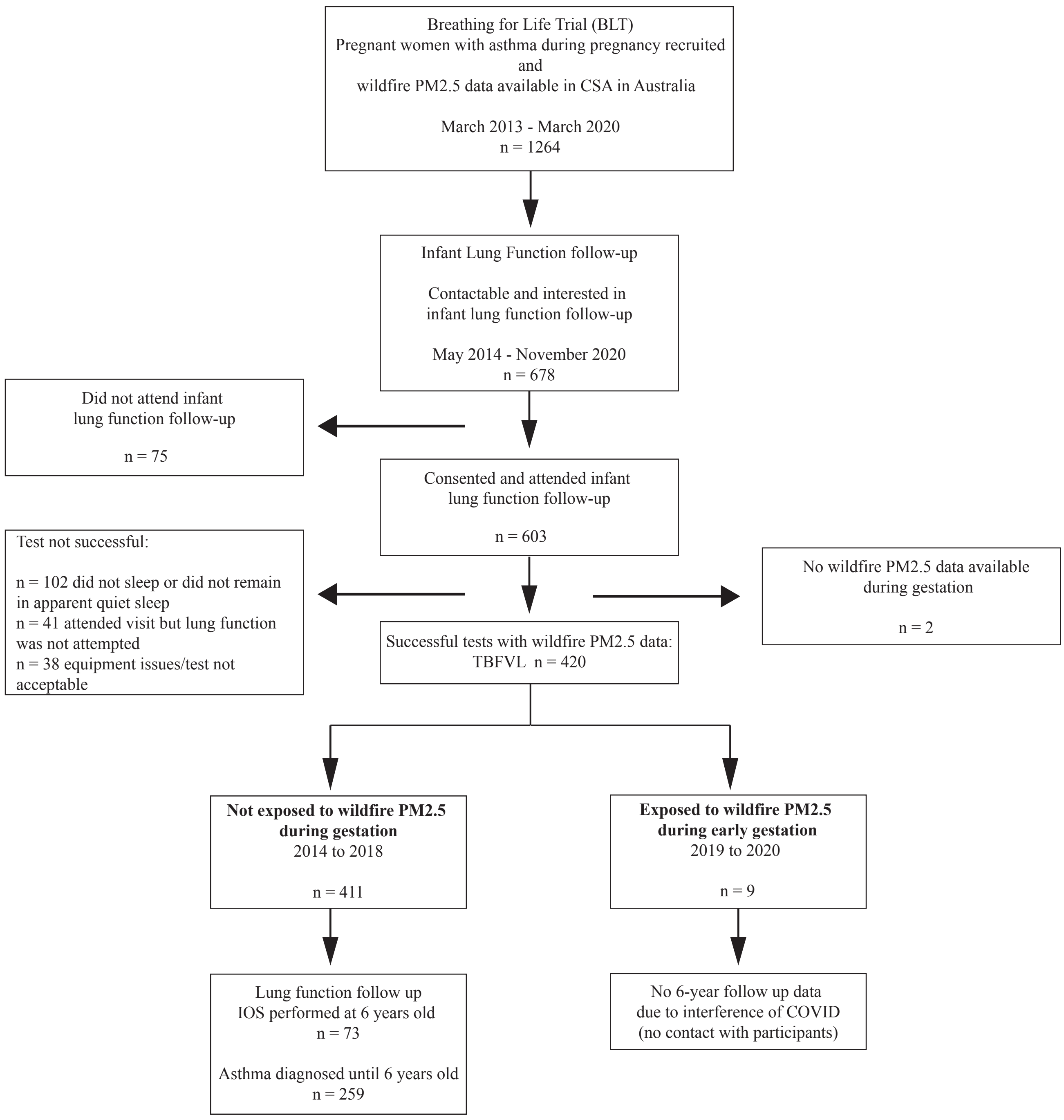

Figure S1. Flow diagram of inclusion and exclusion criteria for the study cohort.

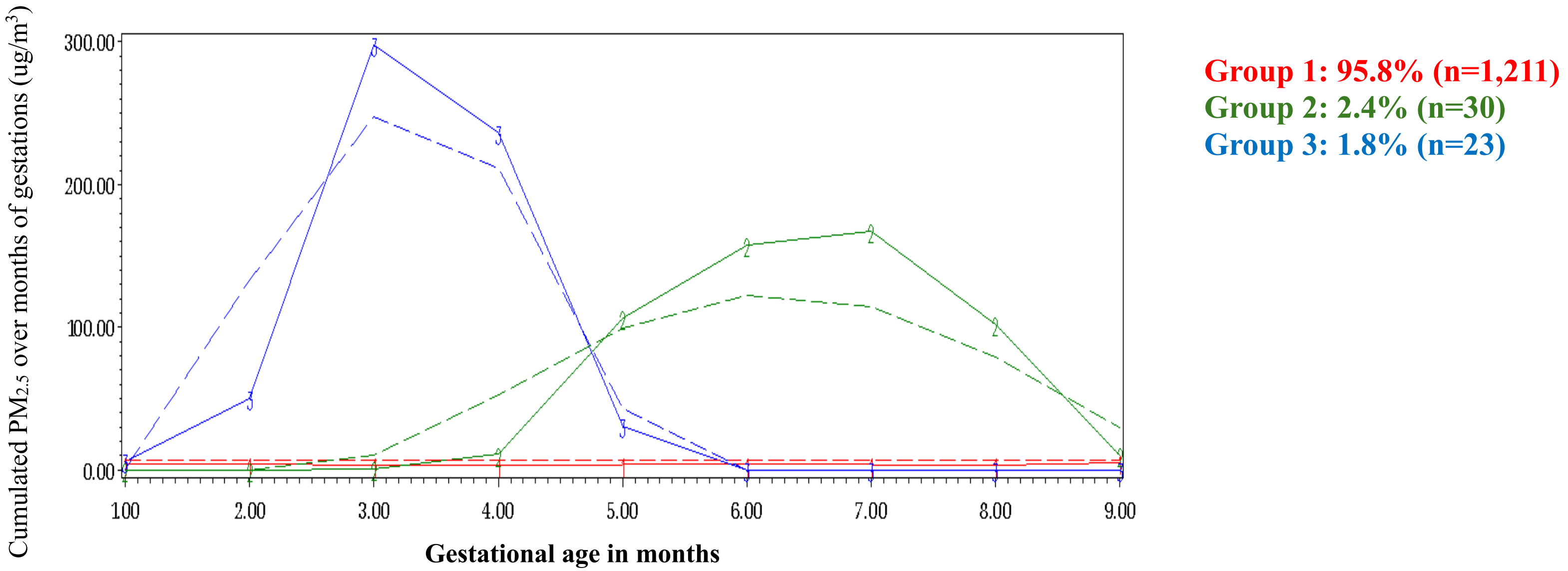

Figure S2: Patterns of cumulative $PM_{2.5}$ exposure from wildfire days by month across the 9 months of gestation.

Table S1. Baseline characteristics of study children at 6 years of age included in an additional analysis of infant lung function and later respiratory outcomes

	6 years IOS assessment and infant lung function data<br>n = 73	6 years ISAAC questionnaire and infant lung function data<br>n = 259
Asthma exacerbation during pregnancy *n (%)*	25 (34.3)	66 (25.5)
Maternal smoking during pregnancy *n (%)*	11 (15.1)	29 (11.2)
Preterm birth *n (%)*	9 (12.3)	22 (8.5)
Caesarean section *n (%)*	24 (32.9)	86 (33.2)
Male *n (%)*	41 (56.2)	138 (53.3)
Twins *n (%)*	2 (2.7)	2 (0.8)
Gestational age at birth in weeks*	38.9 (1.7)	39.1 (1.6)
Birth weight in kg*	3.4 (0.6)	3.4 (0.6)
**Infant assessments**		
Age at infant lung function test in weeks*	6.0 (1.7)	6.6 (1.9)
Weight at infant lung function test in kg*	4.8 (0.7)	4.9 (0.7)
Length at infant lung function test in cm*	55.6 (2.6)	56.0 (3.1)
Breastfed until infant lung function test date *n (%)*	53 (72.6)	192 (74.1)
**6 years assessments**		
Asthma diagnoses until test date *n (%)*	37 (50.6)	116 (44.8)
Age at lung function test in years*	7.2 (0.6)	-
Weight at lung function test in kg*	27.6 (6.4)	-
Length at lung function test in cm*	125.8 (6.5)	-

ISAAC, International Study of Asthma and Allergies in Childhood
*values show mean (SD)

Table S2: Regression analysis to assess the effects of infant lung function on later respiratory outcomes at 6-years old.

	**Rrs at 5Hz** (n = 73) Coefficient (95% CI) p value		**Xrs at 5Hz** (n = 73) Coefficient (95% CI) p value		**Asthma outcome** (n = 259 total, 116 with asthma) OR (95% CI) p value	
	**Crude analysis**	**Multivariable analysis***	**Crude analysis**	**Multivariable analysis***	**Crude analysis**	**Multivariable analysis***
TV, mL	-2.20 (-8.50 to 4.10) 0.489	-0.74 (-6.80 to 5.32) 0.808	3.44 (-6.39 to 13.27) 0.488	0.68 (-8.77 to 10.14) 0.885	1.25 (1.00 to 1.56) **0.048**	2.14 (1.00 to 1.54) 0.051
V'E, mL	-2.58 (-10.16 to 5.00) 0.499	-2.20 (-10.07 to 5.68) 0.580	10.61 (-1.00 to 22.21) 0.073	7.67 (-4.51 to 19.84) 0.213	1.23 (0.90 to 1.69) 0.200	1.15 (0.83 to 1.60) 0.402
MTEF, mL/s	-3.53 (-10.29 to 3.23) 0.301	-3.11 (-9.97 to 3.76) 0.369	4.92 (-5.64 to 15.49) 0.356	2.76 (-7.99 to 13.51) 0.610	1.05 (0.78 to 1.40) 0.759	1.01 (0.75 to 1.36) 0.949
PTEF, mL/s	-1.31 (7.91 to 5.30) 0.694	-0.99 (-7.72 to 5.73) 0.770	6.65 (-3.55 to 16.85) 0.198	5.22 (-5.20 to 15.64) 0.321	1.19 (0.89 to 1.60) 0.234	1.16 (0.86 to 1.57) 0.335
MTIF, mL/s	0.44 (-7.01 to 7.88) 0.907	1.18 (-6.80 to 9.16) 0.769	15.59 (4.57 to 26.61) **0.006**	13.43 (1.43 to 25.44) **0.029**	1.37 (1.03 to 1.80) **0.028**	1.28 (0.96 to 1.70) 0.089
PTIF, mL/s	1.92 (-4.15 to 8.00) 0.530	2.60 (-3.83 to 9.03) 0.422	11.96 (2.88 to 21.03) **0.011**	9.88 (0.10 to 19.67) **0.048**	1.39 (1.10 to 1.76) **0.006**	1.36 (1.07 to 1.73) **0.012**

*Analysis adjusted for sex, maternal smoking during pregnancy, maternal asthma exacerbation during pregnancy, weight at time of infant lung function testing, breastfed until infant lung function testing. Results estimated based on the effect size observed in exposed children. p value < 0.05 in bold.
OR, odds ratio; TV, tidal volume; V'E, minute ventilation; MTEF, mean tidal expiratory flow; PTEF, peak tidal expiratory flow; MTIF, mean tidal inspiratory flow; PTIF, peak tidal inspiratory flow; Rrs, resistance; Xrs, reactance; Hz, Hertz; aOR, adjusted odds ratio.
