# Supplementary figures and images for: Early Gestational Wildfire-Related PM2.5 Exposure Is Associated with Lung Function in Offspring of Mothers with Asthma
Source: Int J Environ Res Public Health. 2026 Mar 3;23(3):314. doi: 10.3390/ijerph23030314 (PMC13026569; doi:10.3390/ijerph23030314)

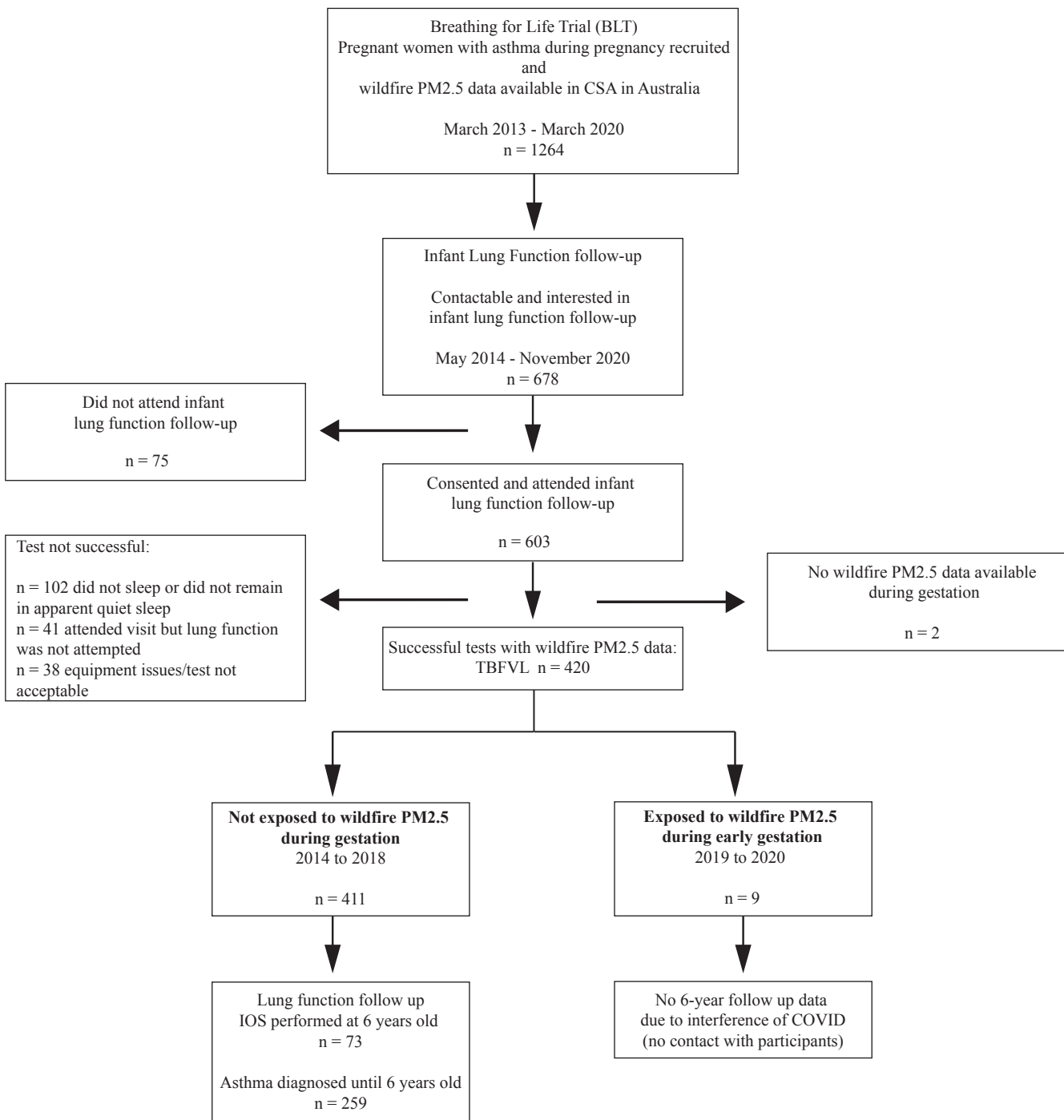

Supplement: Supplementary file 1 [file ijerph-23-00314-s001.zip › supp data/Figure S1.pdf]

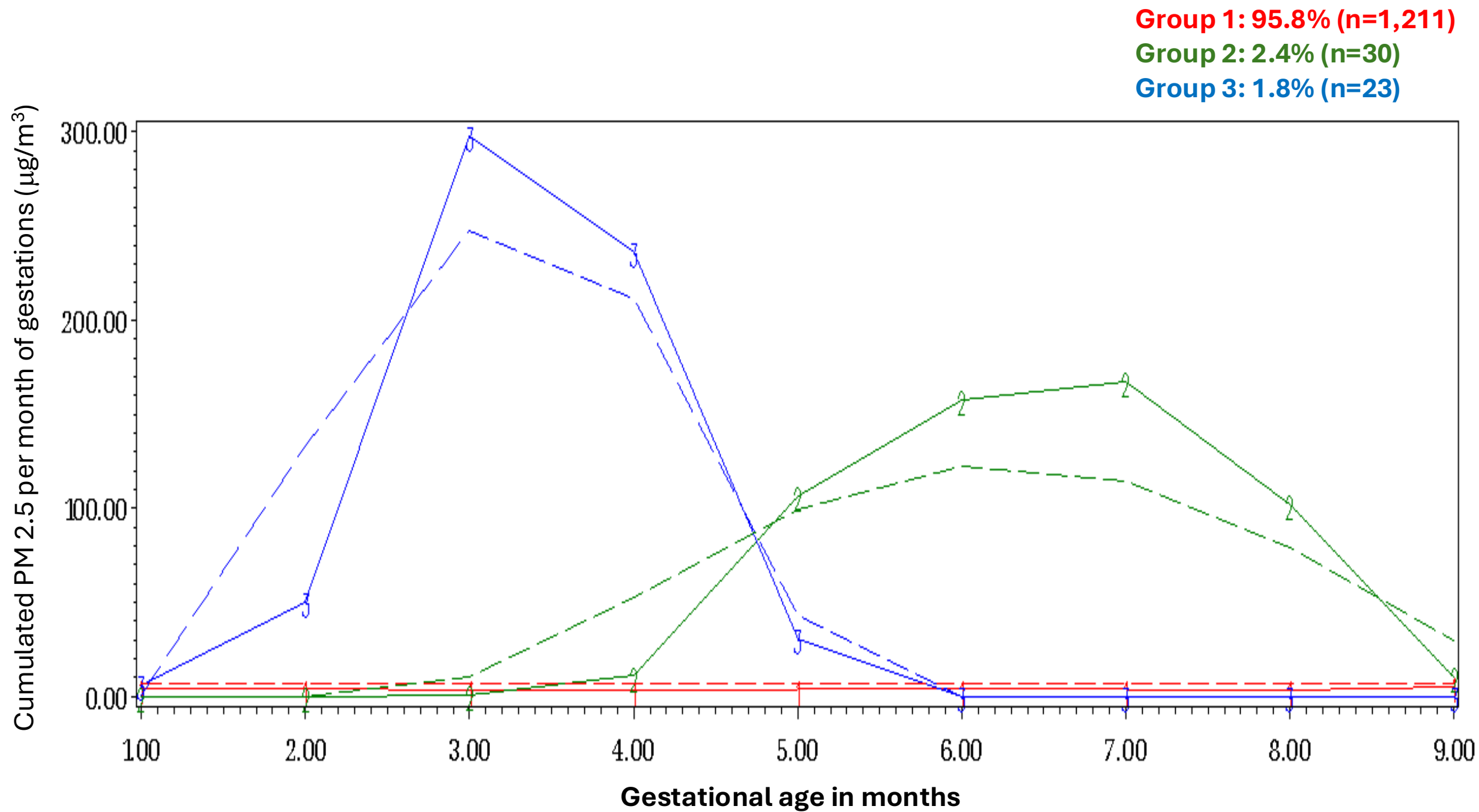

Supplement: Supplementary file 1 [file ijerph-23-00314-s001.zip › supp data/Figure S2.pdf]
